# Station Holding During Rheotaxis: A Sensitive Assay of Lateral Line Function in Larval Zebrafish

**DOI:** 10.21769/BioProtoc.5540

**Published:** 2025-12-20

**Authors:** Sophie Cohen-Bodénès, Elayna I. Malak, Josef G. Trapani, Matt Gaidica, Valentin A. Militchin, Kyle C. Newton, Lavinia Sheets

**Affiliations:** 1 Department of Otolaryngology, Washington University School of Medicine, St. Louis, MO, USA; 2 Department of Biology, Amherst College, Amherst, MA, USA; 3 Department of Neuroscience, Neurotech Hub, Washington University School of Medicine, St. Louis, MO, USA; 4 Department of Biological Sciences, Florida Atlantic University, Boca Raton, Florida, USA; 5 Department of Developmental Biology, Washington University School of Medicine, St. Louis, MO, USA

**Keywords:** Rheotaxis, Zebrafish, Behavior, Lateral line, Machine Learning

## Abstract

Hair cells are the sensory receptors of the auditory and vestibular systems in the inner ears of all vertebrates. Hair cells also serve to detect water flow in the lateral line system in amphibians and fish. The zebrafish lateral line serves as a well-established model for investigating hair cell development and function, including research on genetic mutations associated with deafness and environmental factors that cause hair cell damage. Rheotaxis, the ability to orient and swim in response to water flow, is a behavior mediated by multiple sensory modalities, including the lateral line organ. In this protocol, we describe a rheotaxis assay in which station holding behavior, which employs positive rheotaxis to maintain position in oncoming water flow, serves as a sensitive measure of lateral line function in larval zebrafish. This assay provides a valuable tool for researchers assessing the functional consequences of genetic or environmental disruptions of the lateral line system.

Key features

• Describes the method developed by Newton et al. [1] to assess lateral line function in larval zebrafish.

• Provides instructions on building the micro flume apparatus with updated information from the WashU Neurotech Hub.

• Uses DeepLabCut to track fish and SimBA to classify rheotaxis to compare lateral line–mediated behaviors in large cohorts of larval zebrafish.

## Background

Hair cells function as evolutionarily conserved sensory receptors within the auditory and vestibular systems of the vertebrate inner ear. These same cells act as sensory receptors for detecting water flow in the lateral line systems of amphibians and fishes [2–4]. In the lateral line system, bundles of hair cells, supporting cells, and innervating neurons located near the surface of the skin along the head and the body constitute functional units, called neuromasts, which detect hydrodynamic stimuli (changes in water velocity and turbulence) and sense low-frequency water flow. The hydrodynamic stimuli are transduced by hair cells from a mechanical stimulus into electrochemical signals. Hair cells release the excitatory transmitter glutamate onto afferent neurons of the lateral line ganglia, and the directional flow information is integrated in the hindbrain (lateral line nucleus and cerebellum), along with other sensory modalities interpretable by the central nervous system [3]. These features of sensory hair cell function are highly evolutionarily conserved, and their fundamental mechanisms for transducing sensory stimuli are similar across vertebrate species.

The zebrafish lateral line is a well-established model system for studying hair cell development and function, including studies evaluating the effects of mutations linked to deafness or environmental insults that damage hair cells [5–11]. In larval zebrafish, lateral line hair cells are located superficially along the skin, making them more accessible for experimentation than those of the mammalian ear, which are enclosed in the skull. This accessibility enables the use of various pharmacological treatments and imaging techniques, allowing researchers to apply reagents and visualize cellular processes in an intact larval fish. Yet despite the widespread experimental use of the zebrafish lateral line system in studies of hair cell sensory organ development, damage, and regeneration, there is currently no standard behavioral assay used to evaluate zebrafish lateral line function.

Rheotaxis—the ability to orient into and maintain position in water flow against oncoming current—is a behavior mediated by multiple sensory modalities, including visual, vestibular-tactile, chemotactile, and proprioceptive [12–14], and it includes the lateral line system. Rheotaxis helps fish maintain their position in the water flow and align their body upstream (i.e., positive rheotaxis [2,15]). A specific class of neuromasts, termed superficial neuromasts, is located on the surface of the body and has been shown to mediate rheotaxis [13]. Researchers have developed various methods to study rheotaxis in larval zebrafish, including measuring alignment in a rectangular flume [15], using computer vision to analyze swim patterns in radial flow [14], testing lateral line responses in a suction chamber [16], and employing a line actuator in a horizontal tube to reveal the role of the lateral line in detecting direction [17]. However, none of these approaches has been adopted for general use in laboratories.

Here, we describe a protocol and outline the essential components to reproduce our experimental rheotaxis assay, providing a validated behavior setup to study lateral line–mediated rheotaxis behavior in larval zebrafish. We sought to develop a behavioral assay and analytical methodology that is sensitive, spatiotemporally scalable, and robust enough to be adapted for use on a variety of species. The protocol incorporates computer vision and machine learning tools to precisely quantify the movement, velocity, and acceleration of the fish during rheotaxis. This assay provides the sensitivity needed to quantitatively assess the functional impact of both moderate and severe genetic or environmental disruptions of the lateral line system. For example, our assay has been used in prior studies to define lateral line–mediated behavior deficits in larval zebrafish with a mutation that impairs synaptic neurotransmission at the hair cell synapse [18] and in fish treated with both moderate and high doses of the ototoxic chemotherapy drug cisplatin [1,19].

**Figure 1.**
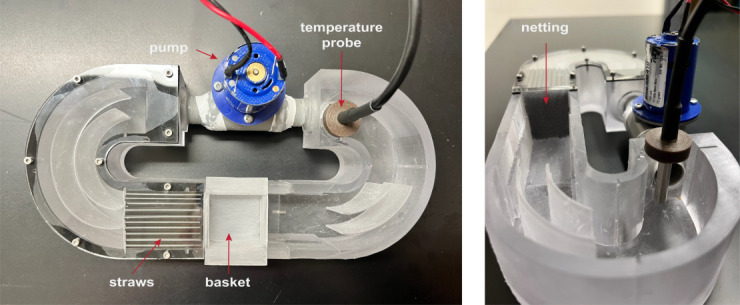
Updated microflume design. Photographs of the latest microflume configuration. (Left) Top-down view showing key components, including the pump/thruster motor (used to generate flow), a straw array (to promote laminar flow), the basket (to contain the zebrafish), and a temperature probe [used to monitor heat generated by the infrared (IR) light source]. In previous setups, a thermometer was manually placed in this location to track temperature. (Right) Side view of the microflume, highlighting the netting that secures the straw array and contributes to uniform flow distribution.

**Figure 2.**
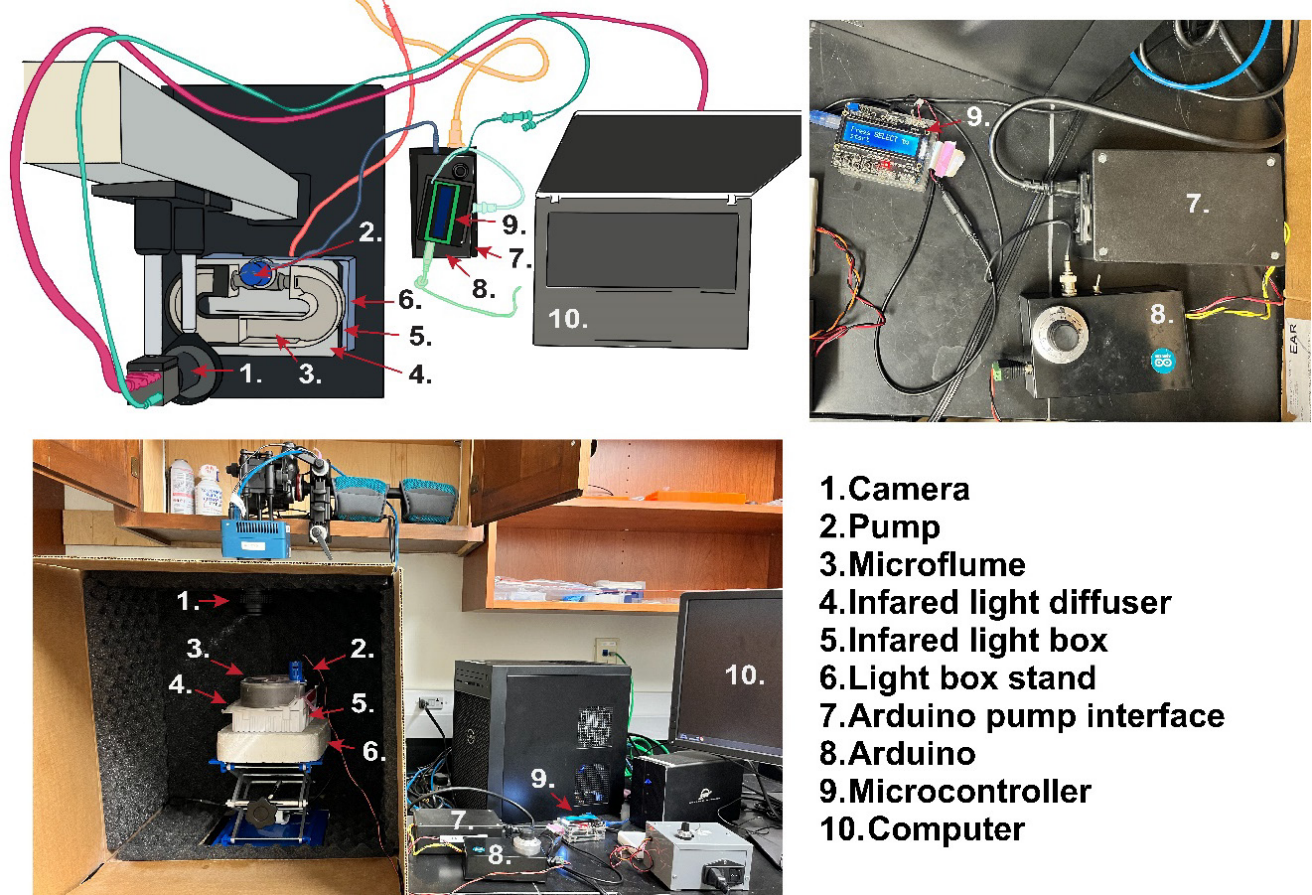
Rheotaxis rig setup. (Top-left) Example schematic diagram of the rheotaxis experimental setup at Amherst College, illustrating possible equipment layout and connections if using an optical rail with a camera and an infrared (IR)-filter and the platform for mounting a light source and the microflume. Components are labeled with numbers corresponding to the legend used consistently across all panels. Different components and their connections are color-coded for clarity: Magenta connection from camera to computer, teal connection from camera to Arduino Interface/Display, blue connection from micro flume pump to Arduino, coral connection from infrared lightbox to power strip, light green connection from Arduino controller to computer, aqua connection from Arduino to Arduino controller, and yellow connection from Arduino to power strip. (Top-right) Close-up view of the Arduino power supply (7), the Arduino and motor/driver, PWM (8), and the Arduino controller (9). (Bottom) Complete view of the assembled rheotaxis rig as implemented at WashU.

Our behavioral setup consists of a translucent 3D-printed microflume with flow collimators providing consistent water flow that is delivered by an Arduino-controlled water pump to a small arena containing a single larval fish. The behavioral arena is illuminated with infrared light from below to eliminate visual guidance cues. The apparatus was developed in-house [1] and has subsequently been adapted and optimized at the Neurotech Hub at WashU (https://neurotechhub.wustl.edu/). We provide the method for printing and assembling the microflume and fish arena/basket (Figure 1) as well as for connecting the hardware and electronics (Figure 2). Furthermore, the flow rate can be parameterized and assayed using our flow velocity assay via visualization with methylene blue under bright light.

This protocol also describes how to process behavioral video recordings and track the fish’s body positions with DeepLabCut. DeepLabCut is a powerful standardized algorithm developed by Mathis et al. [20] that has been extensively used for behavioral tracking of a large variety of animal species, including zebrafish. It is a supervised deep learning tool based on residual networks (ResNet) for feature extraction that requires manual labeling of key body points of the fish, followed by training on a large image dataset (ImageNet), after which it generalizes to automatically track features in datasets of interest.

In addition, we describe the methodological use of SimBA [21], coupled with DeepLabCut, to algorithmically define, extract, and classify rheotaxis behavior. The extracted features are analyzed using customizable R scripts (provided in the GitHub companion to this protocol) to perform spatial, temporal, and spectral analysis, as well as measurements of the fish’s orientation (mean angle vector), velocity, and acceleration relative to the flow. Additional scripts generate heatmaps to visualize the average position occupied by the fish in the chamber arena over time. Time series analysis allows one to quantify both the distance travelled by the fish and the amplitude of oscillations of movements performed to maintain rheotaxis. Spectral analysis further reveals specific patterns of fluctuations in the swimming behavior of the fish to maintain rheotaxis under flow conditions (e.g., the overall trend and fluctuations in linear and angular movement.)

This protocol can be generalized to study other swimming behaviors in fish from different species by adapting the feature extraction script to another behavior of interest, like the C-start or more specific features like the tail posture, or changes in body movement and velocity. Furthermore, by integrating 3D tracking with multiple cameras and extending the size of the microflume, our protocol could be adapted for studying collective behavior, such as shoaling or schooling in multiple fishes.

## Materials and reagents

**Reagents**

1. Methylene blue (Merck, catalog number: M4159-25G)

2. Distilled or nanofiltration water

3. NaCl (Sigma-Aldrich, catalog number: S9888)

4. KCl (Sigma-Aldrich, catalog number: P3911)

5. CaCl_2_·2H_2_O (Sigma-Aldrich, catalog number: C3306)

6. CaCl_2_ (Sigma-Aldrich, catalog number: C1016)

7. MgSO_4_·7H_2_O (Sigma-Aldrich, catalog number: 230391)

8. KH_2_PO_4 _(Sigma-Aldrich, catalog number: P0662)

9. Na_2_HPO_4_ (Sigma-Aldrich, catalog number: S3264)

10. NaHCO_3_ (Sigma-Aldrich, catalog number: S5761)

**Solutions**

1. 1× E3 medium (E3) (see Recipes)

2. 1× embryo medium (EM) (see Recipes)

**Recipes**

*Note: Labs should use in the microflume whatever medium they typically use to maintain larval zebrafish. The following two recipes are routinely used in most zebrafish labs.*

**1. E3 medium (E3)**

**Table:** 

Reagent	Final concentration	Quantity or volume
E3 (60× stock)	1× working solution	1 L
NaCl	5 mM NaCl	34.4 g
KCl	0.17 mM	1.52 g
CaCl_2_·2H_2_O	0.33 mM	5.9 g
MgSO_4_·7H_2_O	0.33 mM	9.8 g
RO or DI H_2_O		

Dilute 16.7 mL of 60× E3 stock in 1 L of reverse osmosis (RO) or deionized (DI) H_2_O for 1× E3 working solution.

**2. Embryo medium (EM)**

**Table:** 

Reagent	Final concentration	Quantity or volume
EM (stock solution)	1× working solution	1 L
1 M NaCl	15 mM NaCl	15 mL
1 M KCl	0.5 mM KCl	500 μL
1 M CaCl_2_	1 mM CaCl_2_	1 mL
1 M MgSO_4_·7H_2_O	1 mM MgSO_4_	1 mL
1 M KH_2_PO_4_	0.15 mM KH_2_PO_4_	150 μL
1 M Na_2_HPO_4_	0.042 mM Na_2_HPO_4_	42 μL
1 M NaHCO_3_	0.714 mM NaHCO_3_	714 μL
RO or DI H_2_O		up to 1L

**Laboratory supplies**

1. Pyrex© media bottle (Merck, catalog number: CLS13951L)

2. Polyethylene transfer pipette, 3 mL (to transfer larval fish) (VWR, catalog number: 16001-188)

3. Multi-well plate (e.g., Costar, catalog number: 3738)

4. Miniature ice packs (Millipore Sigma, catalog number: Z763195)

5. Partial immersion thermometer (e.g., Fisher Scientific, catalog number: 13-201-644) or temperature probe

6. Polyethylene transfer pipette, 1.5 mL, fine tip (to dispense methylene blue) (VWR, catalog number: 14673-010)

7. (Optional) Heat packs 2 × 5 inches with freezable gel (to keep larval fish warm during experiments)

## Equipment

1. Microflume assembly

a. 3D printer (e.g., FormLabs Form 2, 3, or 4)

b. Clear resin (e.g., FormLabs Clear V5 resin)

c. Epoxy (e.g., Loctite Super Glue, liquid clear)

d. Clear silicone sealant (e.g., Loctite Silicone Sealant, clear)

e. Rubber gasket (3M 9448A silicone/rubber, 1.5 mm)

f. Polypropylene straws, 3 mm diameter (e.g., 5'' Sip Stirrer straws)

g. Thruster motor (Raboesch, catalog number: #108-01)

h. Acrylic top plate (TAP Plastics clear acrylic, 3 mm)

i. 88-micron nylon mesh for fish basket and netting in front of flume flow collimators (see Figure 1) (Small Parts Incorporated, CMN-88 A)

2. Arduino UNO R3, Osepp

The Arduino Uno is a microcontroller board based on the ATmega328P chip. It offers 14 digital I/O pins (6 with PWM capability), 6 analog inputs, a 16 MHz clock, USB connectivity, a power jack, an ICSP header, and a reset button. It enables users to build electronics projects by reading sensors, controlling actuators, and interfacing with various components through straightforward programming. Our setup uses 2 units: one to set the pulse width modulation (PWM) and the second with an LCD display to control the triggers for the camera and the pump. The Arduino code is available on the companion GitHub.

3. Digital display for Arduino, LCD display 16 × 2 Osepp

4. Inline rheostat (10k, 10-turn potentiometer with MC33926 Motor Driver Carrier) (Pololu item, catalog number: 1212)

5. Infrared (850 nm) light emitter (e.g., IR 130 LED IR ILLUMINATOR CMVision IR180-198 model: CM-IR130)

6. Diffusion barrier for IR light (Figure 2, component 4; three Kimwipes in a heat seal bag or light diffusing acrylic cut to the dimensions of the light source)

7. Blue light or broad-spectrum light emitter (for flow rate measurements with methylene blue)

8. Light-box stand and lift table (Figure 2, component 6)

a. WashU: a Styrofoam cooler lid (~8'' × 8'') cut to hold the IR emitter on top of an OESS Lift Table Aluminum Oxide Lab Stand Lifter Scientific Scissor Lifting Jack Platform 8'' × 8''

b. Amherst College: Custom 3D-printed light-box stand; optical rail for camera adjustment instead of moving the light-box stand.

9. Camera

a. WashU: high-speed camera (SC1 without IR filter, Edgertronic.com) with a 60 mm manual focus macro lens (Nikon) and 64 GB SD cards

b. Amherst College: Ximea MQ013MG-ON

10. A small screw (3–5 mm long) to substitute for a larval fish when focusing the camera

11. Tripod or optical rail for camera (Amherst College: Thorlabs XT66 66 mm Optical Construction Rail)

12. Computer

a. WashU: Windows 10 operating system based on a Dell Precision 3630 workstation with an Intel Xeon E-2246G processor, 64 GB RAM, multiple 2TB SSD hard drives, EVGA GFORCE RTX 2080Ti video card (GPU), dual 24'' 4 K monitors, Dell Thunderbolt 3 PCIe Card, OWC Mercury Elite Pro Dock (TB3RSDK24T), 24TB Thunderbolt 3 Dock and Dual-Drive RAID configured as 12TB RAID 1

b. Amherst College: ThinkPad T14 laptop computer running Windows 10 (64-bit) with 14-inch UHD (1,920 × 1,080) display and an i5 quad-core processor with 16 GB memory and a 512 GB SSD hard drive

13. Optional: Small LED flashlight (for use during setup of experimental runs in the dark)

14. Optional: Benchtop incubator next to the behavior setup (for housing larval fish during experiment) [WashU: MyTempMini (Benchmark)]

## Software and datasets

1. DeepLabCut2.2b8 (maDLC43,44), Python (3.6 or later), and necessary dependencies (https://github.com/DeepLabCut/DeepLabCut/blob/master/docs/installation.md).

2. SimBA SimBAxTF-development version 68, Python, Git, FFmpeg, and all dependencies (https://github.com/sgoldenlab/simba/blob/master/docs/installation.md).

## Procedure

**A. 3D printing and assembly of the microflume and the testing arena (fish basket)**

The STL files of the microflume components can be found on the companion GitHub repository (https://github.com/sheetslab/rheotaxis) and in the Open Science Framework repository (https://osf.io/rvyfz/).

*Note: The following is a description of 3D printing and assembly of the microflume from the WashU Neurotech Hub*, *https://neurotechhub.wustl.edu/* . *Any readers interested in replicating this protocol should use a similar setup, including an optically translucent microflume, flow collimators to provide laminar flow, a testing arena for the fish larva that allows water flow, and a motor that provides constant water flow.*

The original microflume model was edited in AutoCAD Fusion 360 to enable printing on medium-format resin 3D printers [like the FormLabs Form 2, 3 (with a smaller build volume) or the Form 4 (with a larger ~4–5 L build volume)]. From Fusion 360, the microflume and fish baskets were exported directly to the FormLabs Preform slicer via the print utility export tool (PreForm will also accept raw STL files). Within PreForm, the models were auto-oriented and supported. Finally, the models were printed using FormLabs Clear V5 resin.

After printing, parts were de-supported utilizing flush cutters, micro-pliers, and forceps. Rough edges or critical dimensions were achieved with light filing, sanding, or reaming. The screw holes for the acrylic top plate were hand-tapped with an M2 0.5 mm pitch (standard) tap. All parts were flushed with ethanol (70%–99%) multiple times to wash away debris.

For 3D printers with a smaller build volume (e.g., the Form 2 and 3), the microflume body was divided into two printable pieces with three 3 mm alignment pins to secure both pieces together. The junctions were lightly coated with resin (FormLabs Clear V5) and then pressed together, wiping off any resin overflow using a paper towel or cotton swabs. 3D printers with a larger build volume (e.g., Form 4) can print the entire microflume in one piece. The entire microflume was cured in a FormLabs curing chamber at the recommended settings.

The rubber gasket (3M 9448A silicone/rubber, 1.5 mm) and acrylic top plate (TAP Plastics clear acrylic, 3 mm) were laser-cut via exported DXF files from Fusion 360 using a Boss 2440 105 W CO_2_ laser running LightBurn desktop software and recommended settings. The rubber gasket was washed with warm, soapy water to remove residue from lasing and dried with a paper towel. The rubber gasket was placed between the microflume body and acrylic top plate and secured using standard pitch M2 × 8 mm screws. The Thruster motor (i.e., pump, Figure 1) was positioned in the flume and held in place with Loctite super glue and clear silicon sealant. It was modulated by Aurdino.

For the straw array/flow collimator, 3 mm diameter polypropylene straws were cut to 40 mm in length, placed in the microflume immediately upstream of where the fish basket is placed (see Figure 1), then bound on either side with nylon mesh held in place with Loctite super glue. For the removable fish basket (30 × 30 × 10 mm), pieces of nylon mesh were cut and adhered to the bottom as well as the upstream and downstream sides with Loctite super glue. During use, this removable basket is friction-seated next to the flow collimators (Figure 1).

**B. Rheotaxis rig setup and experiment preparation**

1. (If this is the first run) Assemble the components as shown in Figure 2.

*Note: The PC connection to the Arduino controller (light green connection; Figure 2) is necessary to power the controller and when parameters need to be adjusted. To connect the Arduino to the PC, use a standard USB cable, then install the Arduino IDE software from the official Arduino website.*

2. Prewarm a 1 L bottle of embryo medium (E3 or EM) at 27 °C in the incubator.

3. Rinse the flume and fish basket with distilled water.

4. Turn on the PC and plug in all the equipment as shown in Figure 2 (camera, IR light, Arduino, control boxes). The initial power and speed on the potentiometer in our setup (Figure 2, component 8) is set around 130–135.

5. Place the light diffuser between the flume and light source (see Figure 2, components 4 and 5).

6. Fill the flume with distilled water and use a glass or plastic pipette to remove all bubbles from the fish basket, basket grooves in the flume, and straws (see Figure 1).

**Critical:** Make sure to remove all the bubbles in the flume and fish basket with a plastic pipette, as their presence will affect the water flow rate and may be misidentified as a fish body part and labeled by DeepLabCut. See Troubleshooting section.

7. Connect the camera and check the live camera feed to center the arena in the frame. Allow room for later cropping of video (~20 px border).

a. If using the Edgertronic camera:

i. Connect the camera power, the ethernet cable, and the camera trigger to Arduino, and insert the SD card. Wait for a steady green camera LED and a yellow system LED.

ii. Connect to Chrome; click CAMERA shortcut (10.11.12.13 is the URL).

iii. Click *Camera storage shortcut* (10.11.12.13/storage) to open another tab that will monitor files written to the camera. Refresh this URL/tab to keep track of the trials; it is easy to lose your place.

8. Adjust camera focus by placing a small screw (~3–5 mm long) in the basket and manually focus the camera lens on the threads. This is a good approximation for focusing on live fish.

9. Set up camera settings for optimal exposure (high larval contrast) at 60 fps.

a. Edgertronic camera: 20000 ISO sensitivity, 1/1,000 s shutter, f16, 60fps, 40 s duration.

**Figure 3.**
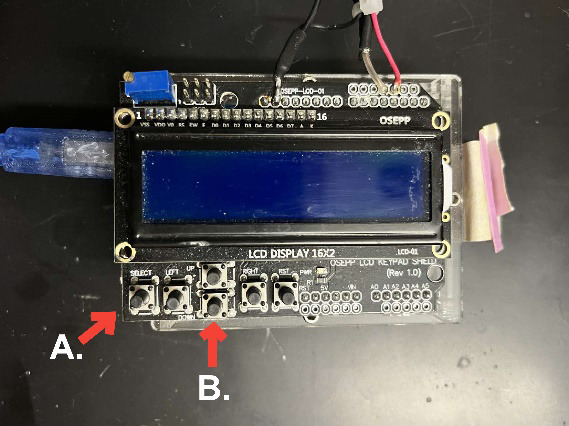
Arduino microcontroller. A: SELECT button; B: DOWN button.

10. Perform test runs without fish to ensure all is working properly.

a. The Arduino microcontroller buttons (Figure 2, component 9; Figure 3) control everything: The first run must press the SELECT button. Thereafter, it is the DOWN button (see Figure 3).

*Note: The Arduino code is available in the companion GitHub.*

b. Run test to confirm whether:

i. The camera acquires video.

ii. The pump starts and stops—verify by observing, by eye or via camera, visible water movement and/or a slight drop in water level within the arena.

iii. A video file is generated.

**C. Running behavior trials**

1. Replace the distilled water in the flume with prewarmed 26–28 °C embryo media (E3 or EM).

2. Use a transfer pipette to remove all bubbles, lint, etc., from the flume.

3. Turn on the IR light and check the live video feed.

4. Monitor the water temperature (26–28 °C) with an alcohol thermometer placed upstream of the pump or with a temperature sensor. If, after multiple trials, the embryo medium moves above 28 °C, then place a small freezer pack just before the pump inlet (see Troubleshooting section) or replace the overheated media with fresh media.

5. The overhead lab lights MUST be turned off, or the setup should be contained within a light-blocking dark box; use an LED flashlight to see fish. If the apparatus is not contained within a dark box, reduce PC monitor brightness to 0. It is important to keep light out of the arena during a trial.

6. Use a transfer pipette to put one fish into the basket/arena. Make sure the fish is actively swimming and exploring the arena. Remove fish that do not show spontaneous swimming behavior or that appear sickly (i.e., listing/laying on its side) as they will not give usable data.

**Critical:** Be sure to remove any debris that may have transferred with the fish from the arena, as it may be misidentified as a fish body part and labeled by DeepLabCut. To avoid the transfer of debris, maintain larvae in clean media prior to the experiment.

7. If the fish is behaving normally, press SELECT/DOWN on the Arduino.

*Note: This setup records 10 s of normal swimming with no flow, then 20 s of rheotaxis under water flow, then 10 s of no flow. To change these parameters, they must be adjusted in the Arduino software. The Arduino file (CamTrig, click Arduino icon on desktop) is a text file, and the relevant line of code that controls the camera and pump timing is: int Initime = 2, CamTrigTime = 1, pumpDelay = 10, pumpOnsec = 20)*

8. Following the behavior trial, remove the fish. If the fish needs to be subsequently genotyped or processed for histology, place it into a labeled individual well of a 24-well plate with prewarmed embryo medium until the experiment is completed. Make note of the video file name and well location for accurate identification during analysis.

9. Place a new fish into the arena and repeat the process (press Arduino SELECT/DOWN). Do not give the fish too much time to stop exploring and just float in place, as it will skew your pre-stimulus control data.

10. Monitor how many fish have been tested for each condition and adjust filenames when switching between conditions. When pausing the experimental trials, turn off the IR light (Figure 2, component 5) to prevent the embryo media from overheating (see Troubleshooting section).

11. After completing the experiment, store all raw video files in a clearly named folder on your computer. This folder will serve as the base directory for subsequent processing steps and downstream analysis.

12. Turn off all the rheotaxis equipment and verify that the infrared light is off (the IR light will overheat if left on for an extended period).

## Data analysis

Files for data analysis are available on the online dataset of Newton et al. [1] and on the companion GitHub repository https://github.com/sheetslab/rheotaxis.

**Overall workflow**

Downsample (make video file smaller) video files in DLC > batch-crop video files in SimBA > analyze video files in DLC > annotate analyzed video files using SimBA > analyze output data in R.

**Automatic tracking with DeepLabCut**

Using DeepLabCut, we obtain pixel coordinates (x,y,z) at specific fish body parts (eyes, swim bladder, tail) at each time point. These coordinates allow us to calculate linear and angular vectors, which define rheotaxis features in our custom feature extraction script.

1. Open DeepLabCut (DLC). This protocol uses the graphical user interface (GUI), which can be launched by running python -m deeplabcut in a terminal.

2. Load the configuration file into DLC (configuration file “config.yaml” can be found in GitHub. Note that prior to this step, you should download the entire DLC folder “1LZF_DLCma_model-Kyle-2020-07-29,” which contains the config.yaml file)

a. Click *Manage Project* tab.

i. Click *Load existing project.*

ii. Select the config file: Click *Browse* and navigate to the location of your config file (e.g., /DLC/1LZF_DLCma_model-Kyle-2020-07-29/config.yaml).

iii. Click *Ok.*

3. Downsample videos (takes ~30 s/video).

a. Navigate to the experiment folder containing all raw videos using file explorer. Create the following subfolders: “Downsampled,” “Cropped,” “avi,” and “raw.” Move all raw video files into a newly created “raw” subfolder.

b. In DLC, click *Video editor* tab.

c. Click *Select video* and load all raw video files (.mov, .mp4, .avi) from your experiment folder.

d. Set “Downsample – specify video height (aspect ratio fixed)” (1,000 in our setup).

e. Click *Downsample*.

f. Drag downsampled videos into the Downsampled folder.

4. Batch-crop videos in SimBA.

a. Open SIMBA GUI.

b. Convert .mov to .avi (takes ~30 s/video).

*Note: If videos are already in .avi format, confirm that videos are readable by OpenCV/check codec compatibility with SIMBA, and skip this step.*

i. Click *Tools* tab > *change formats…*> “change video file format.

ii. In the “Convert multiple videos” pop-up window, select *Browse Folder* > “Downsampled” folder.

iii. Enter “mov” in *Input format*; enter “avi” in *Output format*.

iv. Click *Convert multiple videos*.

c. Drag .avi videos from the “Downsampled” folder into the empty preset “avi” folder.

d. Batch crop the videos in SimBA GUI.

• Click *Tools* tab > *crop video*.

• In the “Crop Video” pop-up window > “Fixed coordinates crop for multiple videos” > “Browse Folder” for video directory > “avi” folder; click *Browse Folder* for output directory > “Cropped” folder.

• Click *Confirm*.

e. A window with a frame from an example downsampled.avi will appear.

• Crop the arena by clicking and dragging.

• Hit *Enter* on the keyboard or exit out of the pop-up window when satisfied (takes ~15 s/video). The software will automatically crop each video according to the fixed coordinates.

5. Analyze cropped videos in DLC GUI interface.

a. Click *Analyze videos* tab in DLC.

b. Configure “Attributes” section to:

• Video type: “.avi”

• Shuffle: 1

• Use ffprobe to read video metadata: yes

• Create video for checking detections: yes

• Tracker method: box

• Overwrite tracking files: no

c. Click *Select videos to analyze* > select all .avi videos in “Cropped” folder. Then, click *step 1: Analyze Videos* (~90 s/video); this will create a .mp4, full.pickle, and meta.pickle file per video.

*Note: DLC GUI will not give a process progress; for progress updates, see the command terminal used to launch DLC.*

d. Review .mp4 videos to confirm that the markerless pose estimation is tracking fish.

e. If acceptable, click *Step 2: Convert to Tracklets*; this will produce a bx.pickle and assemblies.pickle file per video (4 total pickle files per video).

6. Refine Tracklets.

a. Click *Refine tracklets* tab in DLC.

b. Configure the pop-up to:

• Video type: avi

• Number of animals: 1

• Tracker method: box

c. Click *Browse* for “Select the video” and choose the downsampled cropped .avi video.

d. Click *Step1: Create tracks*; this will create a bx.h5 file.

e. Click *Optional: Refine tracks* to view the markerless pose estimation; scroll through the video and adjust the markerless pose as needed.

i. Use the lasso tool to draw a box around the fish to select all markers; unclick the *lasso* tool.

ii. Use the drag tool to relocate the misaligned markerless pose estimator.

iii. Click the house-shaped button to zoom to the original setting.

iv. If a mistake was made, exit without saving and restart.

v. If acceptable, click *save*. This will not confirm that it was saved; an updated time stamp will confirm that the file is saved.

f. Click *Optional Filter Tracks* (then you also get a CSV file) to get a filtered file. This step works only if step e above is completed.

g. Repeat for each individual video; there is no automated way to do this step.

h. Create a new folder called “h5” and drag bx.h5 files into this new folder.

**Critical**: h5 files are used to produce the rest of the analysis data.

**Behavioral feature extraction and analysis with SimBA**

1. Load the project into SimBA.

*Note: Prior to this step, download the SIMBA folder from GitHub.*

a. Prep SIMBA folder for analysis: Navigate to SIMBA > 1LZF_DLCma_model > project_folder. Ensure the video folder is empty. Repeat for the following subfolders of csv: features_extracted, input_csv, machine_results, and outlier-_corrected_movement_location.

b. In SimBA, click *File* > *Load Project* > *Browse File* > *SIMBA* > *1LZF_DLCma_model* > *project_folder* > *project_config.ini*.

c. Click *Load Project*, and a new pop-up window will appear.

2. Import videos to SimBA.

a. Under *Import further videos into project folder*, click *Browse Folder* and choose the folder with the cropped .avi videos.

b. Type “avi” for *File format*.

c. Click *Import multiple videos*; this will copy the cropped videos into the project folder of SimBA.

d. Under *Import further tracking data*, click *File type* and select *H5 (multi-animal DLC).*

e. *No of animals* should be 1, click *Confirm*.

f. *Animal 1 name* will appear below, type “Zebrafish”.

g. Tracking type = “box”.

h. For *Path to h5 file*, click *Browse Folder* to the h5 folder that was made in step 6h from **Automatic tracking with DeepLabCut**.

i. Click *Import h5*; a pop-up with labeled fish will appear. Press *c* if the pop-up is labeled correctly with all 7 labels, or press *x* to display a new random frame; repeat until the pop-up is accurately labeled. Double-click on the zebrafish; press *c* if appropriate, and *x* to restart.

j. Continue until finished with all videos; this will create csv files in the project_folder > csv > input_csv folder; these csv files will be used for analysis in SimBA.

3. Produce csv files for R.

a. In SimBA, click *Video parameters* tab.

b. Click *Set video parameters*. A pop-up with all videos in the project_folder will appear; set fps according to camera fps settings (e.g., 60); set “pixels/mm” on the right to (e.g., “27.66955808”); click *save data*.

c. Click the *Outlier correction* tab and click *Skip outlier correction (CAUTION)*; this will create a csv in “outlier_corrected_movement_location” folder (within project_folder > csv). Wait for the files to generate.

d. Click *Extract features* and check *Apply user defined feature extraction script*.

i. Click *Browse File* > SIMBA > 1LZF_DLCma_model > project_folder > custom_feature_extraction_script > ZebraFish_092229.py.

ii. Click *Extract Features*; this will create a csv in the *features_extracted* folder (within project_folder > csv). Do NOT click *Append ROI data to features (CAUTION)*. Wait for the files to generate.

e. Click *Run machine model* tab.

i. Click *Model Settings* and load “SIMBA > 1LZF_DLCma_model > models > validations > model_files > Rheotaxis_2.sav”.

ii. Set “Threshold” = 0.50 (threshold for rheotaxis); set “Minimum Bout” = 100 (minimum rheotaxis bout length of 100ms).

iii. Click *Set model(s)* and exit out of the pop-up.

iv. Click *Run RF Model*; this will create a csv in the “machine_results” folder (within project_folder > csv). These csv files will be used for analysis in R. Wait for the files to generate.

f. Under the *Analyze Machine Results* subheader, click:

i. Analyze machine predictions; check all boxes (*bout events, total events duration, mean bout duration, median bout duration, first occurrence, mean interval, median interval*) and click *Analyze*.

ii. “Analyze distances/velocity”; confirm 1 animal; select Zebrafish_SwimBladder; set “Bp probability threshold” = 0.5; check “Calculate distance moved within ROI”. Hit *run*.

iii. “Time bins: Machine predictions”; “Set time bine size (s)” = 10 (for 10s/bin).

iv. “Time bins: Distance/velocity”; “Set time bine size (s)” = 10 (for 10s/bin).

4. Backup important files.

a. We encourage backup of the following files/folders:

i. Original .mov files.

ii. Downsampled cropped .avi videos from step 4h.

iii. h5 files from DLC in step 6d.

iv. Input csv files produced in step 7n (input csv files for analysis).

v. csv files produced in step 8e (custom machine model results).

vi. csv files produced in step 8f (standard machine model analysis).

**Data analysis with R script and a custom Python script for feature extraction with SimBA**

1. Install appropriate libraries and packages.

a. Download and open RStudio; then, open the R file appropriate for the type of analysis you want to do (file_name.R)

b. Install the necessary libraries to do analysis and run them: tidyverse, dplyr, circular.

*Note: Additional information on installing R and libraries is available on GitHub.*

2. Description of the R files:

• File 1: "Rheotaxis1_file_prep_concatenate_CuSO4_Neo_Bapta_Shake.R". This R code reads the output h5 data from DLC before importing them into Simba. The loop will concatenate all machine_learning csv files from SimBA into one master file. The output "csv" file from file 1 is the master csv that will be used as the input data file for all forthcoming analyses on the other R files.

• File 2: "Rheotaxis2_circular_data_CuSO4_Neo_Bapta_Shake.R". This R code generates circular graphs corresponding to Figure 3 of [1].

• File 3: "Rheotaxis3_spatial_use_1D_2D_CuSO4_Neo_Bapta_Shake.R". This R code generates spatial heatmaps corresponding to Figure 5 of [1].

• File 4: "Rheotaxis4_Time_Series_anayses_Spectral_decomposition_Fourier_transform_Cross-correlation_ROI_spatial_use_CuSO4_Neo_Bapta.R". This R code generates spectral analysis graphs corresponding to Figure 7 of [1].

• File 5: "Rheotaxis5_SimBA_summary_movt_GLMMtests1.R". This R code performs the statistical analysis (ANOVA and GLMM) and outputs the corresponding p-values.

3. Customize the feature extraction script.

SimBA extracts features (specific characteristics of the rheotaxis behavior) from the pose estimation output obtained after tracking with DeepLabCut. To quantify rheotaxis behavior, we have defined specific features encoding, e.g., mean angle orientation, velocity, and acceleration. Those features are encoded as vectors and compose curated metrics, such as the distance between different labels in the body parts of the fish.

The custom script to be imported to SimBA can be found in this repository under "ZebraFish_092229.py". You can customize this script in Python by editing it to your own behavioral analysis needs.

4. Running the analysis with the R codes.

a. Script R1: The goal of this R script is to create a master csv file of all your rheotaxis data collected in a set of experiments.

i. Create the master.csv file from which you will run your analysis with script “R1_master_csv_prep_xxx.R”.

ii. Create a working folder and create an empty folder called “60 fps”. Copy-paste the machine_results .csv files into this folder.

iii. Optional: Rename the file names to add the specific genotype at the end of each file.

b. Script R2 – R3- R4- R5. Below are specific editing revisions that should be made to the R analysis scripts to run the code with your own master csv file:

i. Adjust the data column names after running the master csv file.

ii. Edit the data type according to your column names, e.g., *master.all&Individual* <- *as.factor(master.all$Individual)* for a column named *"Individual"*.

iii. Reconstitute the selection of columns for the aggregate. The aggregate corresponds to the columns you want to select and stack together to perform your analysis. Each number (1, 2, 3...) is associated with the corresponding column, e.g., c(1,3,5,6,9:13,17,18) is an aggregate that selects columns 1, 3, 5, 6, 9 to 13, 17, and 18.

1) Edit all column names that do not fit your own column names, e.g., "Rheotaxis", "Genotype", "Treatment". Delete, for example, the column "Treatment" if you do not have any specific treatment in your setup.

**Optional:** Edit the name of the variables to fit your own experimental setup, e.g., we used "Cu" as a variable referring to copper sulfate treatment to ablate neuromasts [1].

2) Edit after each "$" to fit your column names, e.g., *$Genotype*.

3) Change the names of the variables in "....", e.g., *Genotype = "mutant"*. Change the "..." according to your own genotype as named on your csv file columns (e.g., *Genotype="wildtype"*).

c. Plotting the graphs. At the end of each file, the included code enables direct plotting of the graphs and their export as PDF files. Edit the color line to have a graph in three colors instead of two.

**Flow velocity assay with methylene blue**

The objective of this assay is to assess the velocity of the flow in the basket of the flume. We typically set the flow to 5–10 mm/s (1–2 body lengths per second).

**A. Flow velocity experimental setup and assay**

1. Remove the IR light emitter and replace with the blue light or bright light emitter.

2. Make note of the rheotaxis video acquisition settings/parameters used during the previous behavioral assay for your own record and replace with new settings for recording with the high-speed camera. Under blue light, Shutter = 100/Sensitivity = 25000.

3. Set the camera parameters: Set frame rate (fps) to 60 or 100 fps (100 fps is easiest to calculate). It will allow you to calculate the time in seconds it takes to the dye front to reach the end of the flume by counting the number of frames captured. The length of the basket is 30 mm. The fps used in the calculation is the one used to record the videos (e.g., 100 fps), not the playback speed.

4. Turn the dial on the inline rheostat to the lowest setting and manually turn it up to allow it to run. Slowly increase the dial until the water has a visible flow under the camera. Make a note of this dial setting as the lowest and first trial setting. Increase dial setting incrementally for subsequent trials.

5. Use the trigger to start both the recordings and the pump in the microflume; drop methylene blue using a transfer pipette (1.5 mL; fine tip) at the edge of the mesh net when flow begins. Put at least 4 drops distributed along the edge between the straw array and the basket.

6. Repeat for every subsequent dial setting.

**B. Analysis of the flow rate with our custom Python script**

*Note: The custom python script and the excel file are available in GitHub*.

1. Click on the video and choose *open with*. Select Windows Media Player.

2. Watch through the mp4 videos generated by the script.

3. Choose a blue dye front that is dark and clear to track.

4. Record the frame number when the dye front is at the front of the basket and when it reaches the back of the basket relative to the fluid flow.

5. Right-click on the video and choose "open with". Select Windows Media Player.

6. Inside the video, right-click and hover over "enhancements".

7. Click on the playback speed settings to watch the video more slowly and correctly record the frames for the dye front.

8. Use the Excel file to calculate the mm/s.

9. Repeat for five videos and compute the average of flow rate.

## Validation of protocol

This protocol has been used and validated (i.e., identified both subtle and severe impairments in lateral line function) in the following research articles:

• Newton et al. [1]. Lateral line ablation by ototoxic compounds results in distinct rheotaxis profiles in larval zebrafish. *Commun Biol.* 6(1): 84. https://doi.org/10.1038/s42003-023-04449-2

• David et al. [18]. Kif1a and intact microtubules maintain synaptic-vesicle populations at ribbon synapses in zebrafish hair cells, *J Physiol*. 2024 Oct 7:10.1113/JP286263. doi: 10.1113/JP286263

• Lee et al. [19]. Evaluation of Cisplatin-Induced Pathology in the Larval Zebrafish Lateral Line, *Int J Mol Sci*. 2022 Nov 18;23(22):14302. doi: 10.3390/ijms232214302.

## General notes and troubleshooting

**General notes**

Our protocol can be generalized to study other swimming behaviors in fishes from different species by adapting the feature extraction script to another behavior of interest, like the C-start or more specific features like the tail posture, movement, and velocity.

**Troubleshooting**

**Problem 1:** The pump does not start.

Possible cause: If the pump does not start, it is likely that the rheostat that is in line between the controller and pump needs to be adjusted. This dial controls the voltage to the pump and changes the flow rate.

Solution: Sometimes you must increase the voltage to start the pump, then back it down to a reasonable value so that it will not blow the fish against the back grate of the basket. Aim for about 5 mm/s (~1 body length/s). You can check this with a multichannel pipettor with 5 μL of methylene blue. The dye should take 5–6 s to cross the 30 mm of the basket.

**Problem 2:** Bubbles affect the flow rate/prevent the pump from running.

Possible cause: Bubbles form when filling the flume with embryo media and are also generated by the pump.

Solution: The water level should be very close to the top, but not spill over when the pump is on.

a. Use a transfer pipettor to blow off bubbles in grooves and inside the basket.

b. Use your finger to remove bubbles on the bottom of the fish basket/arena insert.

c. Make sure there is NO spilled water on the IR light (it messes up video contrast).

**Problem 3:** Water in the flume is too hot.

Possible cause: After 30 min to 1 h of behavior trials, the water becomes too hot for the fish in the microflume due to heating from the IR light source.

Solution: Monitor the temperature constantly with a thermometer placed inside the media in the microflume. When it reaches the critical threshold, place miniature ice packs around the flume. Additionally, take a 5–10 min break and turn off the IR light to let the water cool down. Reset the flume with new media if it gets too hot and the ice packs do not cool it sufficiently.

**Problem 4:** Special precautions: When rinsing, be careful not to saturate the top part of the pump with water, as it may disrupt the electronic components.
